# Impact of Anatomical Variability on Sensitivity Profile in fNIRS–MRI Integration

**DOI:** 10.3390/s23042089

**Published:** 2023-02-13

**Authors:** Augusto Bonilauri, Francesca Sangiuliano Intra, Francesca Baglio, Giuseppe Baselli

**Affiliations:** 1 Department of Electronics, Information and Bioengineering, Politecnico di Milano, 20133 Milan, Italy; 2 Faculty of Education, Free University of Bolzano-Bozen, 39042 Brixen, Italy; 3 IRCCS Fondazione Don Carlo Gnocchi ONLUS, CADITER, 20148 Milan, Italy

**Keywords:** functional near-infrared spectroscopy, magnetic resonance imaging, multimodal imaging, sensitivity estimate, Monte Carlo simulation, anatomical variability

## Abstract

Functional near-infrared spectroscopy (fNIRS) is an important non-invasive technique used to monitor cortical activity. However, a varying sensitivity of surface channels vs. cortical structures may suggest integrating the fNIRS with the subject-specific anatomy (SSA) obtained from routine MRI. Actual processing tools permit the computation of the SSA forward problem (i.e., cortex to channel sensitivity) and next, a regularized solution of the inverse problem to map the fNIRS signals onto the cortex. The focus of this study is on the analysis of the forward problem to quantify the effect of inter-subject variability. Thirteen young adults (six males, seven females, age 29.3 ± 4.3) underwent both an MRI scan and a motor grasping task with a continuous wave fNIRS system of 102 measurement channels with optodes placed according to a 10/5 system. The fNIRS sensitivity profile was estimated using Monte Carlo simulations on each SSA and on three major atlases (i.e., Colin27, ICBM152 and FSAverage) for comparison. In each SSA, the average sensitivity curves were obtained by aligning the 102 channels and segmenting them by depth quartiles. The first quartile (depth < 11.8 (0.7) mm, median (IQR)) covered 0.391 (0.087)% of the total sensitivity profile, while the second one (depth < 13.6 (0.7) mm) covered 0.292 (0.009)%, hence indicating that about 70% of the signal was from the gyri. The sensitivity bell-shape was broad in the source–detector direction (20.953 (5.379) mm FWHM, first depth quartile) and steeper in the transversal one (6.082 (2.086) mm). The sensitivity of channels vs. different cortical areas based on SSA were analyzed finding high dispersions among subjects and large differences with atlas-based evaluations. Moreover, the inverse cortical mapping for the grasping task showed differences between SSA and atlas based solutions. In conclusion, integration with MRI SSA can significantly improve fNIRS interpretation.

## 1. Introduction

Functional near-infrared spectroscopy (fNIRS) is gaining relevance in the imaging of cortical activity in a variety of tasks [1,2,3,4]. Compared to functional MRI (fMRI), it allows for the performance of low-cost and ecological measurements of brain activity in an open environment, also minimizing discomfort to participants. Furthermore, fNIRS provides a direct estimation of both the concentration changes in oxygenated ${\mathrm{HbO}}_{2}$ and deoxygenated hemoglobin $\left(\mathrm{HbR}\right)$, hence providing deeper insight to the hemodynamic response to neural activation compared to the blood oxygen level-dependent signal of fMRI. Moreover, fNIRS is well-suited to multimodal integration with other neuroimaging techniques such as EEG [5] and MRI [6,7].

Acquisitions are based on a grid of optodes alternating near-infrared light sources and detectors, whose neighboring pairs form a measurement channel. However, fNIRS’ practical advantages and noninvasiveness requirements for clinical applications must face two major technological issues: (i) the need of accurate signal processing to reduce artifacts and other confounding factors of physiological origin [8,9,10,11]; and (ii) the fairly large source–detector distance needed to reach the cortex; hence limiting the spatial resolution of fNIRS. The present study addresses the latter issue by focusing on an fNIRS system with optodes placed according to the international 10/5 system [12]. In this case, the large source–detector distance (about 3–4 cm) has a lower spatial resolution compared to the structure of gyri, sulci, and functional areas to be investigated, thus supporting a wider application of methods integrating the fNIRS measures with the subject specific anatomy (SSA) of the cortical surface from an anatomical MRI scan.

An analysis of the potential impact of SSA integration is based on two working hypotheses: (i) the average sensitivity of channels may show sharp drops with cortical depth and lateral displacements [13,14]; (ii) the gyri and sulci pattern over cortical surface may cause a large subject-specific variability [15]. As result, the coupling between a cortical area and a channel may experience large variations, which should be considered when referring channel-wise fNIRS data to cortical areas. 

The specific aim of this study is to quantify in a group of young healthy adults the impact of the above channel sensitivity drop and SSA influence. More generally, the driving reason is to highlight the potential impact of fNIRS integration with the SSA. Indeed, in clinical neurological studies, fNIRS might show easier applicability, particularly in longitudinal studies, compared to fMRI, which remains the actual gold-standard in functional evaluations [16]. Moreover, in neurological patients, an anatomical MRI is routinely available, thus permitting the SSA extraction and the fNIRS improved interpretation. 

Several studies already address the problem of estimating fNIRS channels vs. cortical sensitivity (i.e., forward problem) using analytical approaches [17,18], or (as performed in the present study) using Monte Carlo simulations [19,20,21], which require the computation of the subject-specific optical profile. Additionally, the inverse problem of mapping the surface measures onto the cortical surface has intensively been studied [20]. Within this line of research, other studies have investigated methods for the optimal placement of optodes [22,23,24,25], and the performance of high density diffuse optical tomography (HD-DOT) systems to obtain reconstructions of cortical activation with spatial resolution comparable to fMRI [26,27,28,29,30,31,32].

Nevertheless, fNIRS–MRI integration is not systematically employed in clinical research and practice. Hence, in this study, we employ a standard 10/5 optode placement focusing on the forward problem addressing the factors influencing fNIRS sensitivity profile with respect to individual anatomical variability. Namely, a set of Monte Carlo simulations of light propagation according to MRI SSA is performed in a group of healthy young adults. We propose a numerical method to quantify the sensitivity profile with respect to channel displacements and scalp cortex distance (Section 2.3) and the coupling between channels and cortical parcellations of interest (Section 2.4). We also compared SSA results to the atlas-based anatomy (ABA) of three major atlases, which are often used in cases where the SSA was not available [19,33].

## 2. Materials and Methods

### 2.1. Dataset and Experimental Set-Up

At IRCCS Fondazione Don Carlo Gnocchi, 13 healthy young adults (6 males, 7 females, age 29.3 ± 4.3) underwent both fNIRS acquisitions and anatomical MRI scans. The experiment was approved by the IRCCS Fondazione Don Carlo Gnocchi ethical committee, and all volunteers provided their informed consent. An expert neuroradiologist examined each MRI to exclude the presence of any pathology.

Anatomical MRI scans were acquired using a 3T Siemens Prisma scanner and included a high-resolution, T1-weighted 3D image (MPRAGE, resolution = 0.8 × 0.8 × 0.8 mm^3^), which was processed using the Freesurfer package to extract scalp, skull, cerebrospinal fluid (CSF), grey (GM), and white matter (WM) surfaces.

**Figure 1:**
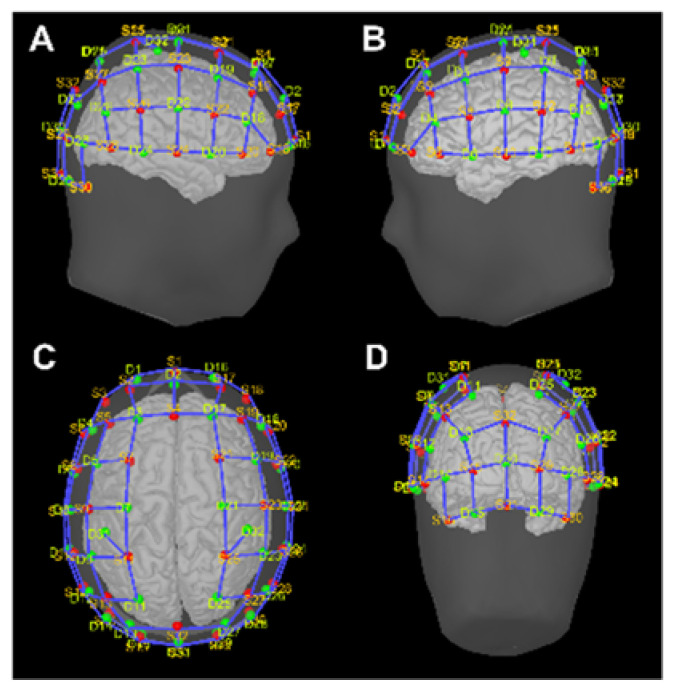
(**A**–**D**) Graphical representation of NIRS source (S, red dots) and detector (D, green dots) positioning over scalp surface ((**A**)—right hemisphere view; (**B**)—left hemisphere view; (**C**)—superior view; (**D**)—occipital view).

The focus of this study is on the analysis of the forward problem and the impact of SSA variability, ahead of the regularization implied by the inverse solution. Nonetheless, application examples of fNIRS cortical mapping are shown on representative subjects performing a motor task. Data were acquired using a continuous-wave system at λ= 760 nm and λ= 850 nm wavelengths (NIRScoutX 32 × 32, NIRx Medizintechnik, Berlin, Germany) that employs 32 LED sources and 32 avalanche photodiode detectors placed according to the international 10/5 EEG system (Figure 1). Source and detector pairs were combined into a total of 102 measurement channels. 

The functional task consisted of a block-designed motor-grasping task, where participants had to alternatively move their left or right hand [8]. The fNIRS signals were preprocessed using the Nirstorm package (https://github.com/Nirstorm/nirstorm (accessed on 5 February 2023)) in Brainstorm [34] to extract the relative changes in oxygenated $\Delta \left[{\mathrm{HbO}}_{2}\;\right]$ and deoxygenated $\Delta \left[\mathrm{HbR}\right]$ hemoglobin concentrations, denoising, and artifact removal. Namely, fNIRS data were firstly preprocessed to remove channels with a coefficient of variation greater than 10%; raw intensity signals were converted into optical density variations, corrected for motion artifacts using the temporal derivative distribution repair (TDDR) algorithm [35], and bandpass-filtered at (0.01–0.08) Hz.

The fNIRS protocol required 40 to 45 min on average, including the placement of the optodes, quality control of channels, and signal acquisition during the considered task, while the MRI protocol average 15 min required on. The pre-processing of fNIRS and MRI data were not time consuming. Conversely, a significant computational burden is referred to—the sensitivity matrix computation over SSA (Section 2.2). Nonetheless, the overall experimental procedure is suitable for clinical applications since the computation of the sensitivity matrix can be performed offline after a routine anatomical MRI is acquired. Moreover, the sensitivity matrix can be reused for several fNIRS trials in a follow-up protocol in most cases where a stationary anatomical condition can be assumed.

### 2.2. Sensitivity Matrix Computation

Anatomical MRI data were processed in Nirstorm to compute the forward model at both wavelengths λ:

(1) $$\Delta OD^{\left(\lambda \right)}=A^{\left(\lambda \right)}\Delta \mu_{a}^{\left(\lambda \right)}$$

Matrix $A^{\left(\lambda \right)}$ elements represent the cortical surface-to-channel sensitivity coefficients at both wavelengths. The channel vector of optical density data is $\Delta OD^{\left(\lambda \right)}=\left[y_{i}\right]$, with i=1, 2, …, I and *I* = 102 number of channels in our configuration. The vector $\Delta \mu_{a}^{\left(\lambda \right)}=\left[{\Delta \mu }_{\alpha ,j}^{\left(\lambda \right)}\right]$, j=1,2,…,J represents the absorption coefficient changes associated to the $j^{th}$ cortical element $\left[{\mathrm{CE}}_{j}\right]$ activity, which in turn is related to hemoglobin concentration changes as ${\Delta \mu }_{\alpha ,j}^{\left(\lambda \right)}=\alpha_{{\mathrm{HbO}}_{2}}^{\left(\lambda \right)}\Delta \left[{\mathrm{HbO}}_{2}\right]+\alpha_{\mathrm{HbR}}^{\left(\lambda \right)}\Delta \left[\mathrm{HbR}\right]$, where the α are the molar absorption coefficients of the respective chromophores at wavelength λ. The solution of this linear algebraic system is hence transferred from the surface channel level to the level of cortical elements $\left[{\mathrm{CE}}_{j}\right]$. Importantly, the $\left[{\mathrm{CE}}_{j}\right]$ are automatically defined in Freesurfer by a vectorized mesh representation of about 15,000 elements for both subject-specific and atlas-based anatomies. 

The optodes were placed on the scalp at the subject’s MRI-native coordinates and a virtual 10/5 cap model was co-registered to the specific anatomy, assuring the correspondence of the relevant landmarks. The resulting grid of sources (red) and detectors (green) is shown in Figure 1.

For the sole purpose of a quality check, all scalp locations and optode landmarks were registered to the Montreal Neurological Institute (MNI) space, thus observing the low dispersion of the clusters of corresponding optodes through subjects and atlases (see Supplementary Material for details).

Matrix $A^{\lambda }$ was estimated using Monte Carlo (MC) simulations in the native MRI space of SSAs, or in the atlas coordinates for ABAs. The forward problem was approached with GPU-accelerated MC simulations [36] with $5\times 10^{7}$ photons per optode. A 5-layer head model was considered: scalp, skull, CSF, GM, and WM. The optical parameters shown in Table 1 were set from the work of Tak et al. [37] and Eggbrecht et al. [27]. Next, the channel-wise sensitivity profile was obtained from the respective source and detector fluencies [38], hence interpolated onto the pial surface (i.e., interface between GM and CSF, from now on simply referred to as cortical surface) using a Voronoi-based method [39] and smoothed with a 2 mm FWHM gaussian kernel.

The same analysis was applied to three atlases. This ABA approach had the dual aim of assessing the validity of applications in which the absence of an individual SSA is surrogated by an ABA [19,33] and also to provide three benchmarks to our SSA group dispersion analysis. The selected atlases were Colin27 [40] due to its wide adoption in fNIRS applications [14] and software [7,41,42], ICBM152 [43] as a standard MNI template, and FSAverage as the default brain template implemented in the Freesurfer package [44].

A qualitative view was provided by mapping the total sensitivity (TS) of all channels vs. cortical elements ${\mathrm{CE}}_{j}$. This was readily computed by summing the values in each column of matrix A:

(2) $$TS^{\left(\lambda \right)}=\left[TS_{j}^{\left(\lambda \right)}\right]=\left[\sum_{i=1}^{I}A_{i,j}^{\left(\lambda \right)}\right]$$

The inverse problem (i.e., projecting surface measures onto the cortex) is ill-conditioned and undetermined, thus requiring heavy regularization. For the given application examples (see Section 3.4), we employed the solution based on the minimum norm estimate approach (for both λ, dropped for clarity), according to the work of Machado et al. [24]:

(3) $$\hat{x}={\left[A^{T}\left({\hat{h}}_{1}C_{1}\right)A+\left({\hat{h}}_{2}C_{2}\right)\right]}^{-1}A^{T}{\left({\hat{h}}_{1}C_{1}\right)}^{-1}y$$

where $\left({\hat{h}}_{1},{\hat{h}}_{2}\right)$ are regularization hyperparameters estimated through the restricted maximum likelihood method, while $\left(C_{1},C_{2}\right)$ are the covariance matrices, respectively, employed to model measurement noise and the a priori distribution of absorption changes.

Notably, Equation (1) indicates the size of regularization to solve for the ill-conditioned and undetermined problem presented in Equation (3), since estimating an order of 10.000 unknown cortical elements’ values from an order of 100 surface measures. Conversely, the present study focuses the forward sensitivity matrix A itself, based on the sole SSA (or atlas) and the simulated optical properties. Namely, two aspects are considered: (i) the drop in sensitivity with lateral displacement, statistically analyzed across channels and subjects by sensitivity displacement surfaces (SDS, Section 2.3); (ii) the variability of sensitivity coefficients grouped by cortical areas into area to channel (A2Ch) sensitivities (Section 2.4).

### 2.3. Sensitivity Displacement Surfaces

This section describes the proposed method to statistically assess the mean 3D profile of sensitivity with respect to displacements from the channel center (CC) at increasing cortical depths. For this purpose, the longitudinal γ (i.e., in the source to detector direction) and transverse τ (i.e., orthogonal direction to γ) curvilinear distances from the CC were defined for each $i^{th}$ channel considering the scalp curvature specific to each subject. The depth d was defined as the distance from a given $\left(\gamma ,\;\tau \right)$ scalp location along the local normal to the scalp down to a cortex element $CE_{j}$, for which sensitivity $A_{i,j}$ was also recorded. Since the cortical element index j is univocally determined by the channel i and the displacement $\left(\gamma ,\;\tau \right)$, the sampled $A_{i,j}$ can be written as $A_{i}\left(\gamma ,\;\tau \right).$ Hence, in a given subject (sbj) and in the frame of the $i^{th}$-channel, a depth map $d_{i}^{sbj}\left(\gamma ,\;\tau \right)$ and a sensitivity map $A_{i}^{\mathrm{sbj}}\left(\gamma ,\;\tau \right)$ were derived. Coordinates $\left(\gamma ,\;\tau \right)$ were sampled over a 40 × 40 mm^2^ field by 2 × 2 mm^2^ bins (with some approximation at borders, due to the curvilinear head shape).

To gain a statistical representation of the sensitivity profile $A_{i}^{\mathrm{sbj}}\left(\gamma ,\;\tau ,d\right)$, depth and sensitivity values from all 102 channels were superimposed by aligning the respective frames. Thus, for each $\left(\gamma ,\;\tau \right)$ coordinate the relationship between depth and sensitivity could be extracted from 102 samples. A segmentation by depth was fixed according to the quartiles of the overall sampled depths in the subject: $d_{Q}^{sbj}$, with the quartile index $Q=\left[1;2;\;3;\;4\right]$ ranging from the least cortical depths (i.e., gyri) to the deepest ones (i.e., sulci). Thus, it was possible to map the average sensitivity profiles at progressive depths according to four sensitivity displacement Surfaces (SDS) averaged across all channels for a single subject:

(4) $$SDS_{Q}^{sbj}\left(\gamma ,\;\tau \right)=\underset{d\in d_{Q}^{sbj}}{\mathrm{mean}}\left[A_{i}^{sbj}\left(\gamma ,\;\tau ,d\right)\right]$$

To quantify the expected drop in sensitivity with depth, the integral value of the four SDS, named integral under the surface (IUS), were computed and expressed in percentage values:

(5) $$IUS_{Q}^{sbj}\left[\%\right]=\frac{IUS_{Q}^{sbj}}{IUS_{1}^{sbj}+IUS_{2}^{sbj}+IUS_{3}^{sbj}+IUS_{4}^{sbj}}\cdot 100$$

where $Q=\left[1;2;\;3;\;4\right]$ indicates the quartile of cortical depth.

The shape of the SDS curves was assessed by the full width at half-maximum (FWHM) along the longitudinal ${\mathrm{FWHM}}_{\lambda }$ and transverse ${\mathrm{FWHM}}_{\tau }$ directions. Namely, ${\mathrm{FWHM}}_{\lambda }$ and ${\mathrm{FWHM}}_{\tau }$ were computed by considering the profile of SDS curves along these directions and calculating the distance between points at half of maximum value. Values for the first and second quartiles of cortical depth are reported, since almost flat SDSs were found at further depths.

### 2.4. Area to Channel Sensitivity

The interpretation of surface fNIRS data are mainly addressed to detect the activations of cortical areas expected to be involved during the execution of a task or in response to specific stimuli. In this perspective, the cortical elements belonging to each area may be grouped as subset of cortical elements $\;CE_{j}$ composing an $area_{a}$ (i.e., cortical parcellation), which are identified by the subset of indexes $J_{a}\equiv \left\{j\;|\;CE_{j}\subset area_{a}\right\}$, with $a=1,\;2\ldots N_{areas}$. Accordingly, the columns of $A^{\lambda }$ relevant to the same $area_{a}$ are summed up into the $a^{th}$-column of a reduced sensitivity matrix $B^{\lambda }$:

(6) $$B^{\lambda }=\left[B_{i,a}^{\lambda }\right]=\left[\sum_{j\subset J_{a}}^{}A_{i,j}^{\lambda }\right]$$

The coefficients $B_{i,a}^{\lambda }$ express the value of area to channel (A2Ch) sensitivities. This reduced model is adopted here to evaluate the sensitivity profile of a parcellation $area_{a}$ due to the specific $i^{th}$-channel and across SSA vs. ABA cases.

The final A2Ch values were normalized using the sum of channel-wise sensitivity coefficients across cortical elements and expressed as percentage ratio.

(7) $$A2Ch\%\left(i,a\right)=\frac{B_{i,a}}{A_{i}}=\frac{\sum_{j\subset J_{a}}^{}A_{i,j}}{\sum_{j=1}^{J}A_{i,j}}$$

As results, $A2Ch\%\left(i,a\right)$ values span in the [0, 1] range and express the relative influence of an area over a measurement channel (i.e., area to channel coupling) and its variability through subjects.

**Figure 2:**
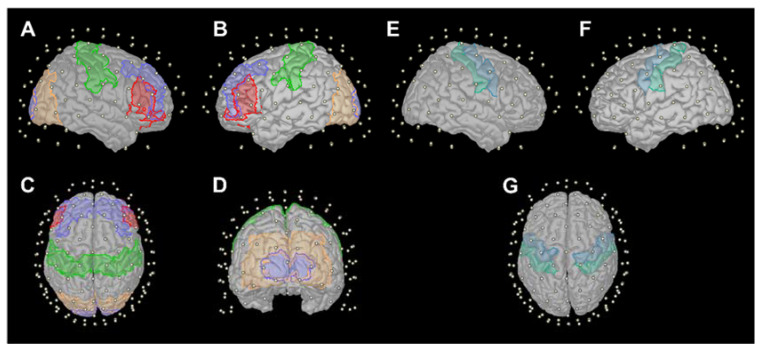
(**A**–**D**) Cortical parcellations according to Brodmann areas on Colin27 atlas: BA1-2-3-4 (green), BA17 (light violet), BA18-19 (orange), BA45-47 (red), and BA46-9 (dark violet). (**E**–**G**) Cortical parcellations according to Destrieux on Colin27 atlas: precentral gyrus (dark blue) and postcentral gyrus (light blue). White dots indicate channel positions over scalp surface.

The A2Ch% values were analyzed on a representative set of Brodmann areas (BA) (Figure 2A–D panels): bilateral BA1–2–3–4 as primary sensorimotor cortex (SensoriMotor Network, SMN), BA17 as primary (Visual Network 1, VIS1), and BA18-19 as secondary visual areas of occipital cortex (Visual Network 2, VIS2), BA46-9 as high-level, and BA45-47 as low-level sites of executive functions of dorsolateral– (PFC1) and ventrolateral–prefrontal cortex (PFC2), respectively. The correspondence between BAs with anatomical areas, along with the notation employed in the results section, is presented in Table 2.

We also considered the bilateral precentral and postcentral gyri as additional anatomical parcellations on the Destrieux atlas [45] (Figure 2E–G panels). Separate analyses of the selected BAs and Destrieux areas were performed. In both cases, A2Ch% values above a threshold of 0.2 are presented and considered as significant channel-wise values (i.e., at least of 20% of the sensitivity profile of the considered area is explained by that channel), while empty channels (i.e., those distant from any considered areas) are omitted.

## 3. Results

### 3.1. Sensitivity Maps

**Figure 3:**
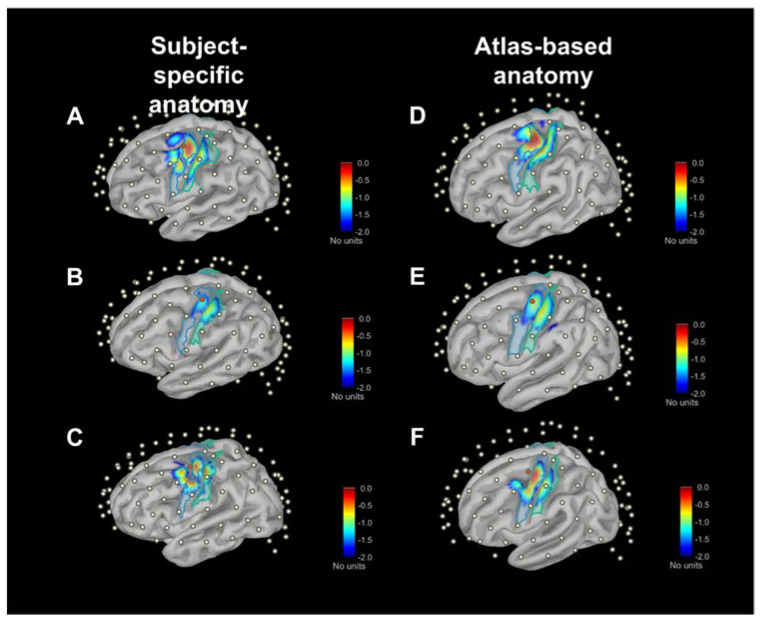
Graphical representation of channel-wise sensitivity profile placed over motor areas (i.e., S9D7) across three SSAs (**A**–**C**) and ABAs ((**D**)—Colin27, (**E**)—ICBM152, (**F**)—FSAverage). The respective cortical parcellations of precentral (dark blue) and postcentral (light blue) gyri are also indicated. Sensitivity values are expressed as $\log_{10}\left(\cdot \right)$ adimensional units. Cortical surfaces were inflated at 40% for visualization purposes.

Figure 3 provides an example of sensitivity profile of channel S9D7 (i.e., the representation of ${\left.\left[A_{i,j}\right]\right|}_{i=S9D7}$ at source 9 and detector 7), while the red dot represents the S9D7 midpoint as channel position. Considering the log_10_ scale down to 1/100 (i.e., blue in the color scale), it is evident that the sensitivity profile drops in a short range of depth from the top of gyri to the bottom of sulci. Moreover, there is a noticeable change in the sensed pattern using SSAs (A–C panels) and ABAs (D–F panels), whose variations can be better-quantified with A2Ch% indexes (Section 3.3).

**Figure 4:**
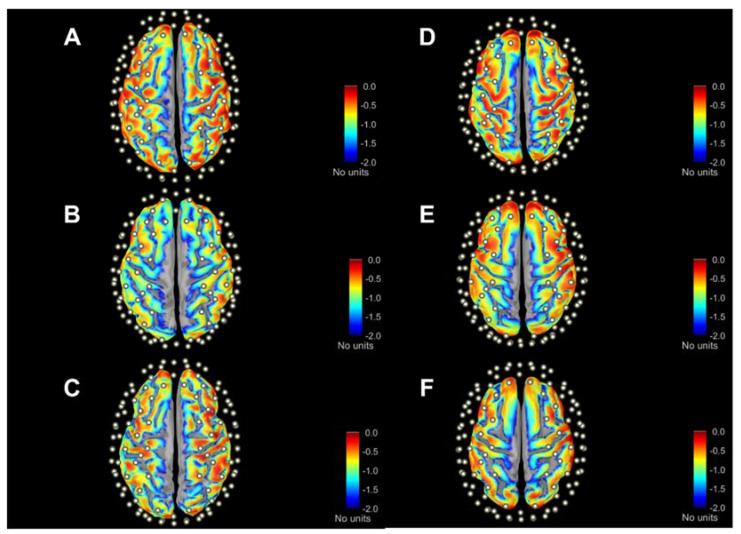
Maps of total sensitivity in three SSAs (**A**–**C**) and ABAs ((**D**)—Colin27, (**E**)—ICBM152, (**F**)—FSAverage) according to anteroposterior direction. Sensitivity values are expressed in $\log_{10}\left(\cdot \right)$ scale. Cortical surfaces are inflated at 40%. All maps refer to λ = 760 nm, while maps for λ = 850 nm are not shown since they display similar patterns.

The total sensitivity $\left[TS_{j}\right]$ maps on the cortical surface are shown in Figure 4 for three subjects and the three atlases. Values are normalized to the maximum sensitivity (i.e., red color scale) and in $\log_{10}\left(\cdot \right)$ scale down to 100-fold less (i.e., blue color scale). The qualitative inspection of these maps anticipates the quantitative results of the following analyses. The most evident feature is that the drop in sensitivity with depth shown above for a single channel (S9D7, as example) is maintained, also summing up all channel sensitivities. This confirms that the signal recorded on the scalp surface is almost entirely determined by the gyri. Secondly, along the sagittal plane, the inflation shows a part of the mesial cortex, which is a gray color scale since it is below the considered sensitivity range, hence negligible. Finally, similarities and differences among SSAs (A–C panels) and among atlases (D–panels) are highlighted. Overall, the maps presented in Figure 3 and Figure 4 attain to MC simulation at λ= 760 nm; however, λ= 850 nm provided similar qualitative information.

### 3.2. Sensitivity Displacement Surfaces

Table 3 provides a summary of the percentage IUS values (median and IQR) at increasing quartiles of cortical depth. Results quantify how the sensitivity profile drops with depth. Additionally, Table 4 presents the depth separation values between each subsequent quartile: $d_{Q1/Q2}$, $d_{Q2/Q3}$, and $d_{Q3/Q4}$, respectively. The ${\mathrm{IUS}}_{1}$ reflects the integral of the sensitivity profile to cortical anatomy at the top of gyri and shows that the sensitivity profile is mostly confined to this cortical range. Values for SSAs had a median of ${\mathrm{IUS}}_{1}\;=$ 0.39, while lower values are found for most ABAs: 0.3 for Colin27, 0.36 for ICBM152. Conversely, the ${\mathrm{IUS}}_{1}$ over the sensitivity profile of FSAverage is comparable to the one of SSAs.

Considering the sum of the two upper quartiles ${\mathrm{IUS}}_{1}+{\mathrm{IUS}}_{2}$ values of 0.683 were found for SSAs, 0.596 for Colin27, 0.656 for ICBM152 and 0.681 for FSAaverage, which further confirms that the top of the sulcogyral pattern should be considered in the interpretation of fNIRS (i.e., almost of 70% of the sensitivity profile is entirely confined within this cortical range). Moreover, Table 4 highlights the steep drop of sensitivity with cortical depth. The quartile separation values in SSAs present a median decrease of about 2 mm per quartile (i.e., 11.8 mm at $d_{Q1/Q2}$, 13.6 mm at $d_{Q2/Q3}$, 15.7 mm at $d_{Q3/Q4}$).

**Figure 5:**
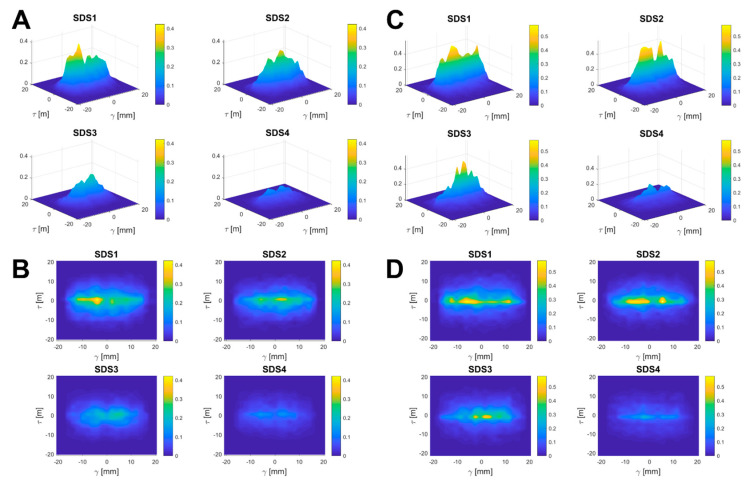
Graphical representation of SDS plots regarding two SSAs at increasing quartiles of cortical depth. (**A**,**B**) Lateral and top views of SDS plots regarding the first subject. (**C**,**D**) Lateral and top views of SDS plots regarding the second subject.

Considering the shape of the SDS plots, it is possible to evaluate the longitudinal and transversal sensitivity decay. Figure 5 displays this analysis relevant to two SSAs. The drop in amplitude with the depth quartile (from ${\mathrm{SDS}}_{1}$ to ${\mathrm{SDS}}_{4}$), which is quantified by the IUS% indexes, is visually confirmed. The sensitivity profile with displacement reveals two major features: (i) high sensitivity is kept for a wide portion of the longitudinal γ displacement, yet with a drop approaching the source and the detector point; (ii) a sensibly steeper drop is seen in the transverse direction τ.

These features were common to all SSAs and are quantified in Table 5 by the full width at half-maximum (FWHM) along the longitudinal ${\mathrm{FWHM}}_{\gamma }$ and transverse ${\mathrm{FWHM}}_{\tau }$ directions, respectively. Values for $SDS_{1}$ and $SDS_{2}$ are reported since they justify most of the sensitivity. The remarks advanced about Figure 5 are confirmed: (i) ${\mathrm{FWHM}}_{\gamma }$ covers about 21 mm of the longitudinal axis, which is about 60% of the source-detector average distance; (ii) the sensitivity profile of SSAs is sensibly confined along the longitudinal direction by the steeper transverse drop with ${\mathrm{FWHM}}_{\tau }$ about threefold narrower than ${\mathrm{FWHM}}_{y}$.

Conversely, this result is not valid for the ABAs case, ${\mathrm{FWHM}}_{\tau ,1}$ and ${\mathrm{FWHM}}_{\tau ,2}$ being almost comparable or even higher than ${\mathrm{FWHM}}_{\gamma ,1}$ and ${\mathrm{FWHM}}_{\gamma ,2}$, respectively. Therefore, this result suggests that ABAs provide a more uniform drop in the sensitivity profile along longitudinal and transverse direction, possibly due to the smoother anatomy resulting from merging many subjects.

### 3.3. Area to Channel Sensitivity

The A2Ch% coefficients are evaluated here, considering the sample areas defined in Section 2.4. Variability is presented by the SSA group statistics (median (IQR) and compared to the values of the three ABAs. Since the result shown in Section 3.2 displayed negligible differences in sensitivities at λ= 760 nm and 850 nm, the following analyses will be shown only for the former wavelength. Table 6 and Table 7 report the results of A2Ch%-coefficients for the selected Destrieux areas, while Table 8, Table 9, Table 10, Table 11 and Table 12 report functional ROIs associated with selected BAs. In both cases, results for SSAs are reported as median (IQR) values across subjects.

To limit the size of these tables, channels displaying an A2Ch%<0.2 in all columns (i.e., SSA median and ABA values) were omitted. Despite this cutoff, the tables show that the considered areas are sensed by a fairly long list of channels, generally around 10. This indicates that the contribution of many channels enters with varying weights the quantification of the activation of a specific cortical area provided by the inverse problem.

Remarkably, in several areas, one or more channels were found with a median A2Ch% close to or above 0.5, hence indicating that those channels prevalently sensed that area. However, in many of these cases, a wide IQR was found. Hence, interpreting that channel as primarily indicative of that area is only worth it for some of the subjects.

SSA results were also compared to ABAs. The tables highlight A2Ch% values for which the SSA median differed by more than 0.2 from any one of the ABA cases in bold. Such differences, when largely present, indicate that surrogating the SSA with an ABA might provide misleading results when mapping surface data to cortical anatomy. Namely, the highest rate of SSA vs. ABA differences (i.e., highest number of highlighted table rows) is found around the VIS regions (Table 8 and Table 9), while the lowest rate around the PFC regions (Table 11 and Table 12). An intermediate case was represented by motor and sensorimotor regions, as highlighted in Table 6 and Table 7 for Destrieux areas and Table 10 for the SMN region. Although the analysis was limited to few representative ROIs, it is worth noting that the IQR range of A2Ch% coefficients across SSAs is often comparable or higher than the median value, hence indicating that there is an intrinsic variability regarding the identification of cortical anatomy.

### 3.4. Example of Cortical Image Reconstruction

**Figure 6:**
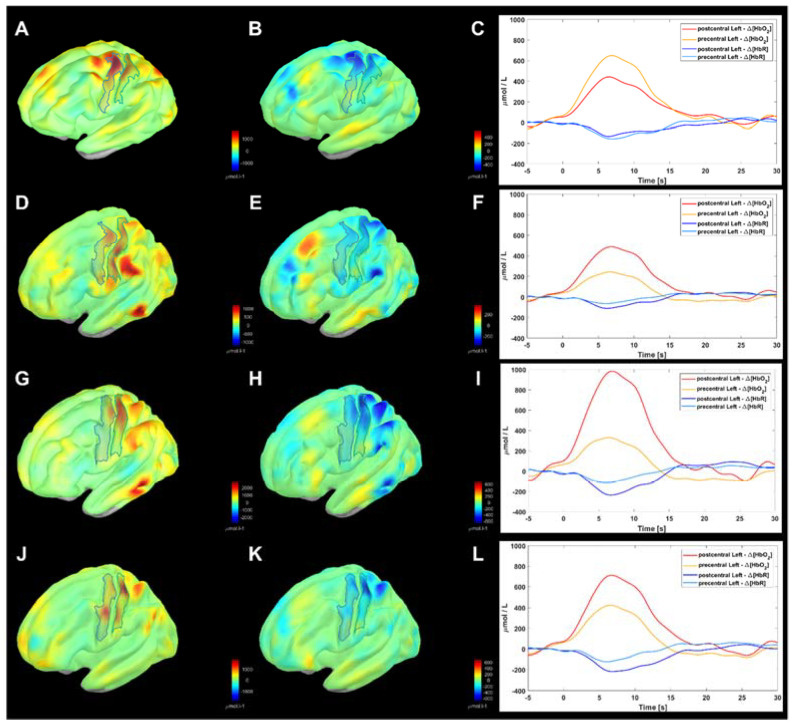
Example of block-averaged image reconstruction in a representative subject (i.e., subject #9) associated to right-hand grasping. From top to bottom: SSA case (**A**–**C**); Colin27 atlas (**D**–**F**); ICBM152 atlas (**G**–**I**); FSAverage atlas (**J**–**L**). From left to right: image reconstruction of $\Delta \left[{\mathrm{HbO}}_{2}\right]\;\mu \mathrm{mol}/L$ concentrations at peak response (**A**,**D**,**G**,**J**); image reconstruction of $\Delta \left[\mathrm{HbR}\right]\;\mu \mathrm{mol}/L$ concentrations at peak response (**B**,**E**,**H**,**K**); mean profile of $\Delta \left[{\mathrm{HbO}}_{2}\right]$ and $\Delta \left[\mathrm{HbR}\right]$ of cortical vertices at precentral and postcentral gyri according to Destrieux (**C**,**F**,**I**,**L**).

Figure 6 provides an example of cortical image reconstruction of the block-averaged precentral and postcentral gyri response to the right-hand motor task in a representative subject (i.e., subject #9). Surface measures, averaged from 10 repetitions, where projected according to the own SSA (A–C panels) and compared with those resulting from employing the three considered atlases: Colin27 (D–F panels), ICBM152 (G–I panels), and FSAaverage (J–L panels). The color maps represent the peak $\Delta \left[{\mathrm{HbO}}_{2}\right]$ and $\Delta \left[\mathrm{HbR}\right]$ values (left and mid column, respectively). The averaged responses are shown in the right column relevant to the contralateral precentral and postcentral gyri (integral in the areas). As expected, the time course shapes were negligibly affected by the adopted anatomy, since they are bound to the task paradigm and to the hemodynamic response function. Conversely, large differences were seen in their amplitudes, in keeping with the differences in the cortical maps.

Namely, the peak of $\Delta \left[{\mathrm{HbO}}_{2}\right]$ response (650 μmol/L) was found in the precentral gyrus if the correct SSA was applied. Conversely, the main activation was shifted on the postcentral gyrus for the ABAs, with large amplitude differences: 450 μmol/L for Colin27, 980 μmol/L for ICBM152, and 700 μmol/L for FSAverage. Importantly, the ratio between the postcentral and the precentral gyrus shows sensible differences, being about 2:1 Colin27 and FSAverage, while up to 3:1 for ICBM152.

This result confirms that the anatomy considered in the inverse problem solution strongly influences the localization and quantification of functional responses, which is explained by the forward problem analyses. Namely, the above results relevant to channel S9D7 can be revisited under this perspective. Qualitatively, in Figure 3, by the sensitivity patterns on different anatomies. Quantitively, by the variable A2Ch% values for the Destrieux left precentral and postcentral gyri in Table 6 and for the L-SMN in Table 10.

## 4. Discussion

In this work, we showed an approach to (i) statistically assess the distribution of fNIRS sensitivity profile with respect to channel displacements and scalp to cortex distance; (ii) quantify the coupling between channels and selected functional/anatomical cortical areas (i.e., A2Ch% sensitivity). The proposed method addressed the multimodal integration of fNIRS and MRI techniques to further support the applicability of fNIRS in clinical research by alleviating the problems related to its limited spatial resolution and indirect sensing of cortical areas. Indeed, clinical applications would require a specific identification of cortical activations in patients with impaired neurovascular activity. The pairing of fNIRS with other recognized clinical neuroimaging techniques, such as anatomical MRI, is essential for progress in this direction while simultaneously taking advantage of its portability and potential applicability in various clinical contexts. For these reasons, our analysis was limited to multichannel fNIRS equipment with optodes placed at 10/5 locations, while the ongoing progresses relevant to more sophisticated instrumentation, optimized optode placement, and/or high-density probes were out of our scope.

Results for SDSs and IUS values provided a numerical and visual outcome regarding the spatial sensitivity distribution of the employed fNIRS probes along longitudinal and transverse direction at varying cortical depths. Results showed the characteristic features of the sensing field in SSAs: (i) along the longitudinal axis (i.e., source–detector direction) the sensitivity profile preserved meaningful values along a high percentage of the entire source–detector distance (i.e., the ${\mathrm{FWHM}}_{\gamma }$ is about 21 mm across both the first and second quartiles of depth), while along the transverse axis we found a steeper decay in sensitivity (i.e., ${\mathrm{FWHM}}_{\tau }$ is about 6 mm across both the first and second quartiles of depth); (ii) a very wide drop in sensitivity is seen with depth, such that the bottom of sulci was attenuated about 100fold compared to the top of the gyri. This last result is quantified by the integral values of SDS plots according to quartiles of cortical depth. Approximately, the first depth quartile (i.e., top of gyri) provided about 40% of the sensitivity, the second about 30%, the third about 20%, and the fourth (i.e., bottom of sulci) about 10%. This result showed limited group dispersion across the SSAs, as quantified by low IQRs, since the *SDS*s were obtained by combining all channels, thus compensating the individual differences in the patterns of gyri and sulci. However, it should be stressed that this result refers to a group of healthy young adults with similar age span. Individual variability of fNIRS sensing with depth may conversely show even wider dispersions incase significant age spans were considered, or even neuropathologies implying brain atrophy [46,47,48].

The comparison of IUS values with the ABAs cases delivered similar results except for the Colin27 atlas, which provided an underestimation of the first quartile. In general, the proposed analysis is in line with recent works. Namely, Tian and Liu [49] proposed ICBM152 as reference for a depth compensation algorithm to improve the localization of functional activation. Liu et al. [50] adopted SSAs, indicating that fNIRS surface measurements can infer deep-brain activity comparable to simultaneous fMRI acquisitions. Strangman et al. [14,51] mapped the depth sensitivity profile over a Colin27 function of source–detector separation using MC simulations and quantified the influence of scalp and skull thickness. Moreover, the interpretation of fNIRS findings can be highly affected by anatomical variability and optode placement [15].

Regarding the A2Ch% analysis, in the fNIRS literature, it is a common practice to constrain scalp measurements to the cortical surface [21,33]. However, to the best of our knowledge, a numerical assessment of the forward problem and individual anatomic variability is usually overlooked, while directly assessing the inverse problem solution and regularization. The analysis was necessarily limited to a few Brodmann areas (BAs), yet representative of frontal, sensorimotor, and occipital regions. Separately, the precentral and postcentral gyri from the Destrieux atlas were also considered, given the different logic of this atlas based on anatomical features. To summarize the huge and sparse intersection between areas and channels, we provided a list of channels for each area, showing A2Ch%≥20%. The major result was the complex portrait of channels sensing each of the considered areas that provided information about the area activation while solving the inverse problem. Importantly, the SSA group analysis revealed high IQRs through the subjects. Finally, for many A2Ch%, the result provided by one or more of the three atlases sensibly differed from the SSA group median. The major deviation of the ABAs from the SSA median were found in the occipital visual regions, decreased in the sensorimotor region, and even more in the frontal region.

So far, fNIRS–MRI fusion is still seldom employed in clinical research and practice. Notwithstanding, the ecological measurements provided by fNIRS could support the evaluation of brain disorders [52,53], functional recovery in stroke [54], epilepsy [55], transcranial electrical stimulation [56], implementations of brain–computer interface and neurofeedback [57,58], monitoring of chronic neurological diseases [59], and the mapping of brain plasticity in rehabilitation [60,61,62].

The results of this study show that the further computational effort needed for cortical fNIRS mapping may correct the nonnegligible individual variability of the sensitivity of channels vs. cortical areas, thus improving interpretation via the inclusion of subject-specific anatomy. Indeed, future developments of this work will addressed the inverse problem of providing a more stable solution by reducing the number of sensitivity matrix coefficients. Namely, the proposed method will allow for the definition of a spatial prior on cortical depth to avoid the inversion of sensitivity matrix elements not significantly contributing to the sensitivity profile (i.e., the SDS analysis of Section 2.3) and/or that do not properly map a cortical area of interest (i.e., the A2Ch% analysis of Section 2.4).

## 5. Conclusions

We reconsidered the current problem of fNIRS image reconstruction by introducing methods for the numerical quantification of the sensitivity profile with respect to cortical depth and coupling with cortical areas of interest. This step requires the estimation of a sensitivity matrix of surface channels vs. cortical elements, whose dimension challenges qualitative and quantitative analyses. We also limited the analysis to a multichannel fNIRS equipment according to a 10/5 system configuration of optodes. The choice was derived from the need for providing concrete indications for the clinical research use of fNIRS. An indication was obtained using an analysis of the cortical surface sensed on average by each scalp channel, which is limited in the transverse direction (orthogonal to the source–detector line) and drops dramatically with depth. The sharp sensing volume and the subjective variability of gyri and sulci, even in a group of young healthy adults, provided high dispersion of the coupling between channels and cortical areas of major interest, as indicated by the area of channel sensitivity coefficients. The analysis performed on three major atlases showed that such standard anatomies may provide a limited surrogate for subject-specific anatomies according to different regions of interest. In conclusion, moving towards the application of fNIRS in clinical contexts would greatly benefit from the integration of fNIRS data with the subject-specific MRI anatomy as routine practice.

## Figures and Tables

**Table 1:** Optical properties of the five-layered model employed in Monte Carlo forward problem (absorption coefficient $\mu_{\alpha }$ scattering coefficient $\mu_{s}$, anisotropy factor g, refraction index n).

	760 nm		850 nm			
	$\mu_{a}$	$\mu_{s}$	$\mu_{a}$	$\mu_{s}$	g	n
**Scalp**	0.017	6.727	0.019	5.818	0.89	1.37
**Skull**	0.0116	8.545	0.0139	7.636	0.89	1.37
**CSF**	0.004	2.727	0.004	2.727	0.89	1.37
**GM**	0.018	7.599	0.0192	6.165	0.89	1.37
**WM**	0.0167	10.825	0.0208	9.188	0.89	1.37

**Table 2:** Summary of employed left (L-) and right (R-) hemisphere areas and their correspondence between Brodmann areas and anatomical areas.

Functional Label	Functional Areas	Anatomical Areas
L-SMN/R-SMN	BA1-2-3-4	SensoriMotor Network
L-VIS1/R-VIS1	BA17	Visual Network 1 (Occipital cortex)
L-VIS2/R-VIS2	BA18-19	Visual Network 2 (Occipital cortex)
L-PFC1/R-PFC1	BA46-9	Prefrontal cortex 1 (Dorsolateral)
L-PFC2/R-PFC2	BA45-47	Prefrontal cortex 2 (Ventrolateral)

**Table 3:** Summary of percentage Integral Under the Surface (IUS) of SDSs at increasing quartile of cortical depth across all channels. Reported values are expressed as median (IQR) across all SSAs.

	λ (nm)	$\mathrm{IU}S_{1}$ (%)	$\mathrm{IU}S_{2}$(%)	$\mathrm{IU}S_{3}$(%)	$\mathrm{IU}S_{4}$(%)
**SSA**	760	0.391 (0.087)	0.292 (0.009)	0.215 (0.035)	0.108 (0.044)
**Colin27**		0.299	0.270	0.261	0.170
**ICBM152**		0.366	0.290	0.228	0.115
**FSAverage**		0.396	0.285	0.210	0.110
**SSA**	850	0.395 (0.089)	0.293 (0.01)	0.214 (0.033)	0.106 (0.043)
**Colin27**		0.300	0.270	0.260	0.169
**ICBM152**		0.367	0.290	0.227	0.115
**FSAverage**		0.400	0.284	0.208	0.108

**Table 4:** Separation values between depth quartiles. Results for SSA case are expressed as median (IQR) across all subjects.

	$d_{Q1/Q2}$ (mm)	$d_{Q2/Q3}$ (mm)	$d_{Q3/Q4}$ (mm)
**SSA**	11.8 (0.7)	13.6 (0.7)	15.7 (1.2)
**Colin27**	14.100	16.100	18.000
**ICBM152**	12.900	14.900	17.100
**FSAverage**	17.200	19.800	22.795

**Table 5:** Full width at half-maximum of SDS plots along the longitudinal (${\mathrm{FWHM}}_{\lambda }$) and transverse direction (${\mathrm{FWHM}}_{\tau }$) at the first and second quartiles of cortical depth. Results are reported as median (IQR) for SSAs case.

	λ (nm)	$\mathrm{FWH}M_{\gamma ,1}$ (mm)	$\mathrm{FWH}M_{\tau ,1}$ (mm)	$\mathrm{FWH}M_{\gamma ,2}$ (mm)	$\mathrm{FWH}M_{\tau ,2}$ (mm)
**SSA**	760	20.953 (5.379)	6.082 (2.086)	21.147 (5.091)	6.187 (4.01)
**Colin27**		29.189	18.331	21.058	24.856
**ICBM152**		28.236	25.034	24.543	20.815
**FSAverage**		18.738	14.108	20.746	21.326
**SSA**	850	20.067 (5.4)	6.082 (1.634)	21.312 (4.718)	6.332 (4.01)
**Colin27**		28.772	18.331	21.058	24.856
**ICBM152**		28.236	25.034	24.543	20.815
**FSAverage**		18.292	14.108	20.523	21.326

**Table 6:** Summary of A2Ch coefficients associated with left hemisphere Destrieux postcentral and precentral gyri. Results are reported for wavelength λ  = 760 nm. Bold ABA values indicate channels presenting a 20% difference in A2Ch coefficients from the SSA median.

Channel	G_postcentralL				G_precentralL			
	SSA	Colin27	ICBM152	FSAverage	SSA	Colin27	ICBM152	FSAverage
S11D31	0.766 (0.4)	0.657	**0.559**	0.953				
S11D7	0.291 (0.417)	0.614	0.516	0.485	0.431 (0.199)	**0.107**	**0.101**	0.462
S11D9	0.682 (0.167)	**0.315**	**0.073**	**0.475**				
S7D7					0.042 (0.121)	**0.309**	**0.431**	**0.318**
S8D5					0.196 (0.218)	0.257	0.272	0.393
S8D8	0.157 (0.133)	0.258	0.253	0.185	0.429 (0.159)	**0.144**	**0.161**	0.402
S9D5	0.022 (0.068)	0.203	0.267	0.057	0.343 (0.232)	0.487	0.542	**0.740**
S9D7	0.121 (0.143)	0.300	**0.623**	**0.244**	0.576 (0.187)	0.567	**0.315**	0.708
S9D8	0.477 (0.191)	0.471	0.297	0.552	0.357 (0.203)	**0.009**	**0.000**	0.312
S9D9	0.689 (0.065)	0.591	**0.198**	0.672				

**Table 7:** Summary of A2Ch coefficients associated to right hemisphere Destrieux postcentral and precentral gyri. Results are reported for wavelength λ = 760 nm. Bold ABA values indicate channels presenting a 20% difference in A2Ch coefficients from the SSA median.

Channel	G_postcentralR				Precentral			
	SSA	Colin27	ICBM152	FSAverage	SSA	Colin27	ICBM152	FSAverage
S21D21					0.04 (0.159)	**0.307**	**0.531**	0.201
S22D19					0.144 (0.154)	0.266	**0.445**	**0.438**
S22D22					0.396 (0.145)	0.233	0.300	0.341
S23D19	0.026 (0.05)	0.069	**0.262**	0.026	0.329 (0.135)	**0.641**	**0.540**	**0.655**
S23D21	0.067 (0.103)	**0.271**	**0.480**	0.082	0.523 (0.237)	0.591	0.479	**0.771**
S23D22	0.461 (0.16)	0.508	0.476	0.504	0.358 (0.129)	0.200	**0.037**	0.332
S23D23	0.599 (0.257)	0.389	0.349	0.679	0.143 (0.086)	0.065	0.004	0.248
S25D21	0.259 (0.23)	**0.562**	0.381	0.355	0.431 (0.184)	**0.201**	**0.090**	0.507
S25D23	0.513 (0.356)	0.322	**0.059**	0.673				
S25D32	0.546 (0.333)	0.730	**0.190**	**0.783**	0.378 (0.457)	**0.023**	**0.000**	0.217

**Table 8:** Summary of A2Ch coefficients associated to selected left hemisphere functional ROIs: primary (VIS1) and secondary visual network (VIS2). Results are reported for wavelength λ = 760 nm. Bold ABA values indicate channels presenting a 20% difference in A2Ch coefficients from the SSA median.

Channel	L-VIS1				L-VIS2			
	SSA	Colin27	ICBM152	FSAverage	SSA	Colin27	ICBM152	FSAverage
S13D12					0.033 (0.069)	**0.406**	**0.305**	0.068
S13D13					0.298 (0.206)	**0.756**	**0.822**	**0.528**
S14D14					0.422 (0.202)	**0.820**	**0.793**	**0.819**
S15D13	0.100 (0.162)	**0.456**	**0.450**	0.168	0.873 (0.195)	**0.544**	**0.550**	0.819
S15D14	0.087 (0.112)	**0.363**	**0.288**	0.165	0.883 (0.189)	**0.637**	0.712	0.835
S15D15	0.527 (0.121)	0.570	0.436	0.417	0.478 (0.142)	0.430	0.564	0.583
S15D30	0.589 (0.311)	0.961	0.971	0.800	0.353 (0.348)	**0.035**	**0.029**	0.165
S16D14					0.988 (0.032)	1.000	1.000	0.997
S16D15					0.973 (0.069)	**0.000**	**0.000**	0.992
S31D15	0.413 (0.229)	**0.269**	**0.000**	**0.113**	0.56 (0.276)	0.731	**1.000**	**0.887**
S31D30	0.729 (0.379)	0.769	0.614	0.480				
S32D13					0.731 (0.128)	**0.963**	**0.972**	0.687
S32D30	0.092 (0.116)	**0.296**	**0.297**	0.128	0.581 (0.37)	0.466	0.392	**0.372**

**Table 9:** Summary of A2Ch coefficients associated to selected right hemisphere functional ROIs: primary (VIS1) and secondary visual network (VIS2). Results are reported for wavelength λ = 760 nm. Bold ABA values indicate channels presenting a 20% difference in A2Ch coefficients from the SSA median.

Channel	R-VIS1				R-VIS2			
	SSA	Colin27	ICBM152	FSAverage	SSA	Colin27	ICBM152	FSAverage
S30D29					**0.982 (0.034)**	**1.000**	**0.000**	**0.992**
S31D29	0.278 (0.122)	0.359	**0.000**	0.226	0.522 (0.251)	0.641	**0.000**	0.769
S32D27					0.611 (0.189)	0.845	0.855	0.798
S27D27					**0.251 (0.178)**	**0.892**	**0.863**	**0.545**
S28D26					**0.022 (0.022)**	**0.383**	**0.251**	**0.297**
S28D28					**0.416 (0.269)**	**0.813**	**0.722**	**0.911**
S29D27	0.156 (0.220)	**0.614**	**0.647**	0.283	**0.825 (0.192)**	**0.386**	**0.353**	**0.714**
S29D28	0.131 (0.148)	**0.372**	0.264	0.204	0.846 (0.167)	0.628	0.736	0.796
S29D29	0.564 (0.173)	0.642	0.509	0.392	0.429 (0.215)	0.358	0.478	0.608
S29D30	0.456 (0.153)	**0.792**	**0.861**	**0.768**	**0.414 (0.374)**	0.046	0.067	0.172
S30D28					0.978 (0.118)	1.000	1.000	1.000

**Table 10:** Summary of A2Ch coefficients associated to selected functional ROIs associated to sensorimotor network (SMN). Results are reported for wavelength λ = 760 nm. Bold ABA values indicate channels presenting a 20% difference in A2Ch coefficients from the SSA median.

Channel	L-SMN				R-SMN			
	SSA	Colin27	ICBM152	FSAverage	SSA	Colin27	ICBM152	FSAverage
S11D31	0.956 (0.256)	0.867	0.891	0.992				
S11D7	0.581 (0.360)	**0.884**	**0.858**	0.787				
S11D9	0.922 (0.189)	**0.586**	**0.348**	**0.641**				
S12D8	0.532 (0.253)	**0.258**	0.345	0.427				
S12D9	0.482 (0.205)	**0.177**	**0.037**	**0.202**				
S8D8	0.210 (0.199)	0.186	0.390	0.245				
S9D5	0.104 (0.188)	0.292	**0.503**	0.241				
S9D7	0.266 (0.276)	**0.726**	**0.869**	**0.628**				
S9D8	0.756 (0.182)	0.877	0.904	0.727				
S9D9	0.913 (0.116)	0.862	**0.486**	0.834				
S22D22					0.227 (0.137)	0.211	0.341	0.156
S23D19					0.081 (0.080)	0.231	**0.380**	0.098
S23D21					0.270 (0.200)	**0.763**	**0.854**	0.340
S23D22					0.743 (0.167)	0.861	0.937	0.611
S23D23					0.904 (0.091)	0.859	0.762	0.877
S25D21					0.555 (0.227)	**0.887**	**0.876**	0.605
S25D23					0.933 (0.106)	**0.589**	**0.464**	0.907
S25D25					0.446 (0.238)	**0.099**	**0.067**	0.430
S25D32					0.863 (0.214)	0.860	0.800	0.870
S26D22					0.701 (0.114)	0.623	0.629	**0.259**
S26D23					0.474 (0.238)	**0.224**	**0.083**	0.350
S27D23					0.280 (0.174)	**0.064**	**0.023**	0.334

**Table 11:** Summary of A2Ch coefficients associated to selected left hemisphere functional ROIs: dorsolateral (PFC1) and ventrolateral prefrontal cortex (PFC2). Results are reported for wavelength λ = 760 nm. Bold ABA values indicate channels presenting a 20% difference in A2Ch coefficients from the SSA median.

Channel	L-PFC1				L-PFC2			
	SSA	Colin27	ICBM152	FSAverage	SSA	Colin27	ICBM152	FSAverage
S4D2	0.521 (0.156)	0.576	0.513	0.375				
S2D2	0.341 (0.25)	0.291	0.363	0.211				
S2D3	0.892 (0.108)	0.892	0.906	0.796				
S3D1					0.402 (0.171)	0.416	0.268	0.355
S3D4	0.257 (0.196)	0.283	0.197	0.239	0.745 (0.186)	0.710	0.797	0.729
S4D3	0.891 (0.174)	0.847	0.774	1.000				
S5D4	0.308 (0.091)	0.290	0.319	0.211	0.692 (0.102)	0.694	0.665	0.782
S5D5	0.638 (0.087)	0.772	0.785	0.452	0.252 (0.077)	0.119	0.078	0.405
S6D4					0.976 (0.096)	0.952	0.985	1.000
S6D6					0.678 (0.177)	0.496	0.634	**1.000**
S7D3	0.619 (0.382)	**0.412**	**0.360**	0.612				
S7D5	0.742 (0.194)	**0.540**	0.582	0.621				
S8D4					0.863 (0.2)	0.767	0.729	0.763

**Table 12:** Summary of A2Ch coefficients associated to selected right-hemisphere functional ROIs: dorsolateral (PFC1) and ventrolateral prefrontal cortex (PFC2). Results are reported for wavelength λ = 760 nm. Bold ABA values indicate channels presenting a 20% difference in A2Ch coefficients from the SSA median.

Channel	R-PFC1				R-PFC2			
	SSA	Colin27	ICBM152	FSAverage	SSA	Colin27	ICBM152	FSAverage
S17D17	0.87 (0.166)	0.862	0.927	0.781				
S17D2	0.284 (0.251)	0.211	0.351	0.204				
S18D16					0.253 (0.176)	0.404	0.297	0.394
S18D18	0.383 (0.163)	0.290	0.185	0.391	0.615 (0.174)	0.698	0.808	0.575
S19D17	0.983 (0.051)	0.946	0.947	0.993				
S19D18	0.356 (0.193)	0.206	0.260	0.330	0.637 (0.184)	0.782	0.727	0.669
S19D19	0.730 (0.072)	0.572	0.699	0.628	0.202 (0.145)	0.250	0.184	0.326
S20D18					0.929 (0.055)	0.935	0.984	0.939
S20D20					0.761 (0.257)	**0.479**	**0.497**	**1.000**
S21D17	0.602 (0.308)	0.453	0.363	0.620				
S21D19	0.619 (0.194)	0.540	0.495	0.711				
S22D18					0.864 (0.145)	0.841	0.808	0.809
S22D19					0.202 (0.164)	0.160	0.105	0.232
S22D20					0.283 (0.196)	0.141	**0.045**	0.312
S23D19	0.416 (0.188)	**0.190**	**0.130**	0.239				
S4D17	0.783 (0.242)	0.754	0.765	0.785				

## Data Availability

The data are not publicly available due to privacy issues.

## Supplementary Materials

The following supporting information can be downloaded at: https://www.mdpi.com/article/10.3390/s23042089/s1.
