# Supplementary material for: Impact of Anatomical Variability on Sensitivity Profile in fNIRS–MRI Integration
Source: Sensors (Basel). 2023 Feb 13;23(4):2089. doi: 10.3390/s23042089 (PMC9962997; doi:10.3390/s23042089)
Supplement: Supplementary file 1 [file sensors-23-02089-s001.zip › sensors-2149778-supplementary.pdf]

Summary of mean MNI locations ($x_{MNI}, y_{MNI}, z_{MNI}$) and RMS Euclidean Distance (σ_{MNI}) of employed measurement channels over scalp surface across SSAs and ABAs (i.e., Colin27, ICBM152 and FSAverage). These locations are identified as mid-position between source (S) and detector (D) positions. The correspondence between source and detector positions with respect to the International 10/5 system is also reported.

Channel-wise $\sigma_{MNI,i}$ values were computed according to the following formula

$$\sigma_{MNI,i} = \sqrt{\frac{\sum_{k=1}^{K}\left(\left(x_{k,i} - x_{MNI,i}\right)^2 + \left(y_{k,i} - y_{MNI,i}\right)^2 + \left(z_{k,i} - z_{MNI,i}\right)^2\right)}{K}}$$

where i indicates the channel index and ($x_{MNI,i}$, $y_{MNI,i}$, $z_{MNI,i}$)) indicate the mean channel-wise MNI locations across $k = 1,2,\ldots,13$ subjects.

channel	reference	SSA				Colin27			ICBM152			FSAverage			ABA
		x_{MNI}I	y_{MNI}	z_{MNI}	σ_{MNI}	x_{MNI}I	y_{MNI}	z_{MNI}	x_{MNI}I	y_{MNI}	z_{MNI}	x_{MNI}I	y_{MNI}	z_{MNI}	σ_{MNI}
**S1D1**	**Fpz - Fp1**	-14.72	84.23	-0.51	3.62	-14.94	85.36	0.14	-13.67	82.56	1.60	-14.77	86.33	-7.06	4.15
**S1D2**	**Fpz - AFz**	-1.01	82.55	21.83	4.87	-0.56	83.72	22.44	0.26	80.98	23.76	-0.92	86.14	15.06	4.40
**S1D16**	**Fpz - Fp2**	13.75	84.56	0.20	3.84	14.17	85.51	0.52	15.33	82.74	2.47	13.74	87.71	-6.34	4.34
**S2D1**	**AF3 - Fp1**	-30.82	77.68	10.42	4.22	-31.13	76.68	10.20	-29.91	74.20	11.13	-31.27	81.16	4.33	4.21
**S2D2**	**AF3 - AFz**	-17.53	77.13	33.31	5.42	-17.51	77.57	33.80	-16.86	75.27	34.68	-17.84	82.70	27.51	4.47
**S2D3**	**AF3 - F1**	-30.58	65.36	44.22	6.03	-31.10	63.97	44.73	-30.78	62.33	45.24	-31.71	70.83	40.24	4.33
**S3D1**	**AF7 - Fp1**	-40.60	74.04	-6.40	3.54	-41.49	74.08	-5.86	-40.07	72.15	-4.60	-41.42	78.06	-11.58	3.96
**S3D4**	**AF7 - F5**	-56.88	56.86	4.87	4.39	-57.69	54.17	4.31	-55.94	52.27	4.60	-57.68	59.20	-0.27	3.77
**S4D2**	**Fz - AFz**	-1.78	67.50	55.16	6.64	-0.79	66.74	55.96	-0.71	64.84	57.15	-1.36	73.82	51.40	4.60
**S4D3**	**Fz - F1**	-14.71	55.49	65.67	7.35	-14.34	53.52	66.96	-14.50	51.41	66.82	-15.14	61.86	63.52	4.80
**S4D17**	**Fz - F2**	11.46	56.40	66.80	7.34	13.57	55.15	68.46	13.27	52.49	68.78	13.02	62.81	64.19	4.86
**S5D3**	**F3 - F1**	-37.76	53.13	53.10	6.76	-38.96	51.00	54.13	-38.74	49.43	53.84	-40.21	59.61	51.31	4.70
**S5D4**	**F3 - F5**	-54.84	49.55	31.38	5.67	-57.63	48.08	32.59	-56.69	46.72	32.25	-58.35	55.22	28.77	4.16
**S5D5**	**F3 - FC3**	-52.49	37.96	49.90	6.93	-54.84	33.87	50.55	-54.75	33.16	50.16	-54.42	40.28	45.57	3.93
**S6D4**	**F7 - F5**	-64.32	45.18	4.18	4.75	-67.99	44.15	4.90	-65.61	42.28	4.62	-67.19	49.07	0.38	3.67
**S6D6**	**F7 - FT7**	-71.33	29.47	-10.79	4.91	-72.12	25.14	-11.82	-70.71	24.84	-11.29	-74.23	31.86	-14.18	3.77
**S7D3**	**FC1 - F1**	-30.07	40.53	69.93	7.92	-30.81	36.41	71.39	-30.95	34.65	70.08	-32.17	45.71	70.02	4.93
**S7D5**	**FC1 - FC3**	-45.25	25.75	67.58	8.00	-47.16	19.95	69.24	-46.99	18.81	67.07	-48.67	29.25	68.40	4.82
**S7D7**	**FC1 - C1**	-34.02	11.41	82.74	9.23	-35.36	3.11	85.71	-36.10	2.33	83.60	-36.90	12.96	86.42	5.02
**S8D4**	**FC5 - F5**	-67.42	34.18	22.06	5.86	-69.03	29.67	20.88	-67.14	28.47	19.86	-68.20	35.37	16.68	3.59
**S8D5**	**FC5 - FC3**	-65.60	22.84	41.16	7.04	-67.68	16.90	40.60	-66.34	16.17	38.73	-67.45	24.14	37.31	3.89
**S8D6**	**FC5 - FT7**	-75.32	18.84	7.44	5.81	-77.96	13.83	6.63	-76.34	13.71	6.20	-78.09	20.45	4.01	3.45
**S8D8**	**FC5 - C5**	-75.76	6.44	28.42	7.23	-76.27	-1.41	25.23	-75.55	-0.99	24.35	-79.07	6.79	25.50	4.10
**S9D5**	**C3 - FC3**	-59.55	9.45	60.19	8.34	-62.62	1.32	60.84	-62.46	1.24	58.89	-64.68	10.53	61.19	4.59
**S9D7**	**C3 - C1**	-49.20	-4.57	77.07	9.60	-51.35	-15.17	78.49	-52.64	-14.97	77.56	-54.06	-5.25	82.06	5.14
**S9D8**	**C3 - C5**	-71.33	-6.65	49.20	8.61	-72.03	-16.38	46.12	-74.16	-15.50	47.02	-75.15	-7.56	48.24	4.27
**S9D9**	**C3 - CP3**	-63.46	-21.75	68.14	10.02	-65.21	-33.68	66.02	-67.14	-32.92	65.85	-69.15	-24.71	71.15	5.01
**S10D6**	**T7 - FT7**	-78.21	2.94	-10.17	5.65	-78.40	-3.64	-12.27	-78.54	-2.75	-11.72	-81.05	3.37	-12.98	3.38
**S10D8**	**T7 - C5**	-80.29	-9.22	11.71	7.18	-80.68	-17.47	8.47	-81.42	-16.38	8.67	-78.91	-10.37	6.51	3.44
**S10D10**	**T7 - TP7**	-80.81	-24.39	-8.36	7.09	-79.60	-32.30	-11.75	-80.93	-31.16	-11.31	-83.17	-25.74	-10.65	3.25
**S11D7**	**CP1 - C1**	-35.60	-20.11	90.27	10.71	-36.35	-33.20	91.08	-38.04	-33.09	89.61	-38.49	-23.42	95.72	5.35
**S11D9**	**CP1 - CP3**	-50.03	-37.38	81.83	11.23	-49.87	-51.52	77.97	-52.33	-51.16	78.04	-53.25	-42.65	84.91	5.42
**S11D11**	**CP1 - P1**	-33.71	-53.56	88.75	11.93	-32.72	-68.47	83.44	-35.07	-68.15	82.53	-34.47	-59.23	88.87	5.21
**S11D31**	**CP1 - CCP3h**	-43.52	-28.39	86.41	11.00	-44.90	-42.72	86.64	-46.94	-42.36	85.88	-46.92	-32.97	90.58	5.06

**S12D8**	**CP5 - C5**	-79.38	-23.34	33.32	9.07	-75.25	-32.53	25.36	-76.90	-31.52	25.64	-81.36	-25.22	30.04	4.66
**S12D9**	**CP5 - CP3**	-72.18	-38.57	53.01	10.32	-68.97	-49.57	44.53	-73.36	-49.56	47.12	-74.87	-42.31	51.75	5.18
**S12D10**	**CP5 - TP7**	-79.84	-38.20	13.16	9.17	-79.44	-48.28	8.54	-79.81	-46.66	7.97	-82.75	-41.17	11.32	3.69
**S12D12**	**CP5 - P5**	-75.34	-54.23	34.69	10.58	-72.61	-64.55	26.99	-73.65	-62.88	26.02	-72.66	-55.77	28.31	3.95
**S13D9**	**P3 - CP3**	-60.43	-54.63	68.22	11.48	-58.34	-66.92	59.47	-61.22	-66.54	59.96	-61.82	-59.03	65.91	4.91
**S13D11**	**P3 - P1**	-43.96	-70.61	74.68	12.42	-41.01	-83.53	63.90	-43.80	-83.50	64.07	-43.36	-75.61	70.67	5.03
**S13D12**	**P3 - P5**	-64.03	-70.54	50.28	11.56	-59.70	-80.86	39.51	-61.72	-79.73	38.84	-65.63	-76.09	48.75	5.54
**S13D13**	**P3 - PO3**	-48.88	-83.82	56.71	12.26	-44.53	-93.01	43.74	-47.06	-92.64	43.53	-48.97	-89.26	54.09	5.52
**S14D10**	**P7 - TP7**	-77.78	-52.26	-3.98	8.84	-75.17	-60.70	-8.98	-76.04	-59.20	-9.12	-80.35	-55.84	-5.44	3.49
**S14D12**	**P7 - P5**	-72.89	-68.31	17.24	10.34	-68.07	-76.61	8.93	-66.29	-72.68	6.46	-75.88	-73.65	16.50	6.20
**S14D14**	**P7 - PO7**	-67.37	-80.68	3.47	10.20	-62.23	-87.00	-4.13	-64.27	-86.25	-4.20	-69.65	-85.98	2.85	4.58
**S15D13**	**O1 - PO3**	-37.00	-103.25	31.49	11.89	-31.84	-106.86	18.02	-33.54	-105.47	17.02	-35.94	-107.12	28.41	5.47
**S15D14**	**O1 - PO7**	-46.42	-102.48	11.48	11.16	-41.34	-106.64	1.45	-42.95	-105.35	0.84	-45.57	-105.68	9.27	4.25
**S15D15**	**O1 - I1**	-32.63	-111.38	-6.93	10.56	-28.49	-113.43	-14.92	-29.79	-111.84	-15.41	-31.74	-114.41	-8.14	3.73
**S15D30**	**O1 - Oz**	-17.42	-112.28	19.83	11.59	-14.38	-116.07	8.21	-16.34	-116.01	7.89	-16.04	-114.97	16.70	4.20
**S16D14**	**PO9 - PO7**	-57.91	-93.07	-15.19	9.99	-52.45	-97.14	-21.14	-54.52	-96.96	-21.17	-58.02	-96.64	-15.92	3.38
**S16D15**	**PO9 - I1**	-44.10	-101.82	-33.65	9.53	-40.30	-105.08	-37.51	-40.91	-102.99	-37.54	-44.11	-105.16	-33.44	2.74
**S17D2**	**AF4 - AFz**	15.69	77.52	34.56	5.67	16.87	77.87	34.56	17.37	74.73	35.69	16.46	83.38	28.56	4.77
**S17D16**	**AF4 - Fp2**	30.22	78.93	12.59	4.77	31.55	79.76	12.61	31.45	73.54	12.88	30.36	81.64	5.44	4.91
**S17D17**	**AF4 - F2**	29.01	66.55	46.46	6.31	31.24	65.66	46.43	31.25	62.11	47.09	30.83	72.12	41.65	4.80
**S18D16**	**AF8 - Fp2**	40.11	75.14	-4.21	4.11	40.96	75.35	-4.36	41.91	71.74	-2.38	40.61	79.24	-10.18	4.54
**S18D18**	**AF8 - F6**	57.19	58.35	7.75	4.80	57.35	55.27	5.52	57.37	50.98	6.88	57.09	60.00	0.83	4.51
**S19D17**	**F4 - F2**	36.04	55.29	55.61	6.89	38.65	52.87	54.62	38.54	49.80	55.57	38.93	61.19	51.84	5.07
**S19D18**	**F4 - F6**	54.70	51.87	34.45	5.97	57.60	50.11	33.32	57.45	46.28	34.32	57.38	56.36	29.13	4.73
**S19D19**	**F4 - FC4**	51.34	40.30	54.07	7.09	55.12	36.82	52.92	54.35	33.13	53.11	55.81	44.95	50.95	5.07
**S20D18**	**F8 - F6**	64.65	46.41	6.87	4.79	68.09	45.93	6.30	67.35	41.14	7.29	67.14	50.33	1.58	4.52
**S20D20**	**F8 - FT8**	72.38	31.21	-7.00	4.48	71.06	26.83	-9.79	71.65	23.46	-7.92	73.93	33.54	-12.03	4.68
**S21D17**	**FC2 - F2**	26.99	42.24	72.92	7.89	30.60	39.22	74.52	29.77	36.10	74.12	30.59	48.12	72.78	5.16
**S21D19**	**FC2 - FC4**	42.52	27.44	71.83	8.05	47.06	22.61	72.49	46.04	19.56	72.42	47.74	31.91	72.50	5.30
**S21D21**	**FC2 - C2**	29.90	12.40	85.58	9.23	34.30	4.68	86.89	33.01	2.37	86.76	34.30	14.06	87.19	5.09
**S22D18**	**FC6 - F6**	67.49	35.82	25.66	5.85	69.72	32.24	23.00	67.64	27.20	22.57	67.56	36.55	17.90	4.58
**S22D19**	**FC6 - FC4**	64.94	24.78	46.14	6.94	68.36	19.95	43.85	67.72	16.50	44.58	69.37	27.79	42.70	4.83
**S22D20**	**FC6 - FT8**	76.04	20.93	12.23	5.29	77.53	16.66	9.35	76.99	12.93	10.49	77.05	22.21	6.25	4.22
**S22D22**	**FC6 - C6**	75.78	8.10	33.18	6.94	76.05	1.40	27.28	80.01	-0.76	32.08	78.28	8.76	27.37	4.92
**S23D19**	**C4 - FC4**	58.01	10.94	65.33	8.34	63.69	4.31	65.01	62.71	1.28	65.44	62.64	11.99	61.99	4.79
**S23D21**	**C4 - C2**	46.09	-3.72	80.94	9.66	50.77	-13.16	79.63	49.33	-15.63	79.55	51.40	-4.07	81.62	5.13
**S23D22**	**C4 - C6**	70.18	-5.41	53.94	8.30	77.11	-13.13	54.11	76.04	-16.24	54.61	73.95	-5.69	49.77	5.10
**S23D23**	**C4 - CP4**	61.18	-20.92	72.27	9.74	66.02	-31.13	68.84	64.07	-33.64	68.18	67.27	-22.87	71.38	4.98
**S24D20**	**T8 - FT8**	79.97	4.80	-5.18	5.05	78.37	-0.60	-9.06	78.71	-3.80	-7.40	80.42	5.44	-10.20	4.10
**S24D22**	**T8 - C6**	81.16	-7.80	16.66	6.60	83.78	-14.10	13.00	83.00	-17.35	13.94	83.69	-7.70	11.80	4.12
**S24D24**	**T8 - TP8**	81.68	-23.24	-3.27	6.36	85.03	-29.79	-6.51	85.02	-32.88	-5.14	82.36	-23.73	-7.80	4.15
**S25D21**	**CP2 - C2**	31.86	-19.79	93.14	10.70	36.51	-31.86	92.78	34.34	-33.59	91.13	36.96	-22.49	96.63	5.52
**S25D23**	**CP2 - CP4**	47.22	-37.07	85.20	11.04	51.01	-49.90	80.40	48.81	-51.70	79.38	52.63	-41.41	86.29	5.65
**S25D25**	**CP2 - P2**	30.35	-53.08	90.92	11.68	34.16	-67.60	86.20	31.20	-67.86	82.80	34.93	-59.33	93.21	6.08
**S25D32**	**CP2 - CCP4h**	40.13	-28.03	89.75	10.87	45.36	-41.12	88.74	43.07	-42.88	87.25	46.23	-32.02	93.43	5.60
**S26D22**	**CP6 - C6**	79.00	-22.07	37.68	8.22	84.56	-30.54	34.91	82.36	-33.40	34.55	78.58	-22.89	29.70	5.60
**S26D23**	**CP6 - CP4**	70.69	-37.74	56.96	9.68	74.76	-48.64	52.30	73.04	-51.13	52.21	73.42	-39.91	51.91	4.87

**S26D24**	**CP6 - TP8**	80.71	-37.74	18.29	8.03	80.50	-45.32	11.78	77.72	-47.39	11.42	81.69	-38.78	13.02	4.09
**S26D26**	**CP6 - P6**	73.96	-53.69	39.03	9.58	73.26	-61.76	29.63	71.35	-63.78	29.46	76.96	-56.20	34.73	4.66
**S27D23**	**P4 - CP4**	57.87	-53.95	71.74	10.88	59.22	-64.45	61.51	58.42	-67.28	63.08	61.05	-57.08	66.92	4.98
**S27D25**	**P4 - P2**	40.91	-69.75	77.20	12.09	42.48	-82.09	66.52	39.85	-82.70	64.78	44.76	-76.06	76.06	6.13
**S27D26**	**P4 - P6**	61.43	-69.97	54.14	10.98	60.12	-78.41	41.70	58.31	-80.17	41.54	64.28	-73.71	50.02	5.42
**S27D27**	**P4 - PO4**	45.38	-83.28	59.00	11.70	44.60	-90.89	44.73	42.81	-92.49	44.74	47.93	-87.50	54.85	5.62
**S28D24**	**P8 - TP8**	77.75	-51.97	0.82	7.79	76.07	-58.20	-5.51	73.83	-59.86	-5.25	79.99	-53.72	-2.57	3.87
**S28D26**	**P8 - P6**	71.49	-68.03	21.67	9.49	70.33	-75.77	13.28	68.14	-77.11	13.02	73.66	-70.64	17.86	4.23
**S28D28**	**P8 - PO8**	66.19	-80.11	7.24	9.23	63.58	-85.49	-1.08	61.19	-86.03	-1.31	68.38	-82.97	4.53	4.25
**S29D27**	**O2 - PO4**	33.70	-102.69	32.98	11.39	32.76	-107.73	20.32	30.72	-108.00	19.72	35.10	-105.89	29.00	4.70
**S29D28**	**O2 - PO8**	44.35	-101.89	13.95	10.50	42.23	-105.49	3.37	40.05	-105.22	2.80	46.53	-106.31	11.88	4.97
**S29D29**	**O2 - I2**	31.15	-110.97	-5.12	9.95	29.38	-112.44	-13.61	27.56	-111.74	-13.86	32.20	-113.61	-6.92	3.82
**S29D30**	**O2 - Oz**	14.70	-111.84	20.72	11.36	14.95	-116.03	9.12	12.78	-113.74	7.60	15.93	-114.89	17.51	4.65
**S30D28**	**PO10 - PO8**	57.11	-92.67	-12.00	8.99	54.75	-97.37	-18.51	52.41	-97.01	-18.45	58.54	-95.40	-14.01	3.40
**S30D29**	**PO10 - I2**	43.98	-101.82	-31.12	8.65	41.39	-103.61	-35.70	39.90	-103.54	-35.19	45.81	-105.51	-31.63	3.23
**S31D15**	**Iz - I1**	-16.10	-114.30	-25.58	9.78	-13.65	-114.66	-31.12	-14.86	-113.69	-31.19	-15.48	-117.27	-25.82	3.03
**S31D29**	**Iz - I2**	15.36	-114.28	-24.69	9.43	14.75	-114.45	-30.47	13.39	-113.80	-30.37	16.41	-117.10	-25.18	3.11
**S31D30**	**Iz - Oz**	-1.00	-115.56	1.24	10.57	0.34	-120.28	-7.33	-1.32	-118.18	-8.16	0.09	-118.33	-0.55	3.61
**S32D13**	**POz - PO3**	-21.73	-96.36	55.55	12.65	-18.47	-105.30	42.06	-20.73	-104.97	41.35	-20.54	-101.74	52.34	5.37
**S32D27**	**POz - PO4**	17.31	-96.19	56.21	12.46	18.04	-104.27	42.05	15.97	-105.18	41.84	18.74	-99.80	50.95	4.99
**S32D30**	**POz - Oz**	-1.92	-104.77	43.45	12.13	-0.02	-110.62	29.64	-2.11	-111.00	29.33	-0.50	-109.18	39.96	5.08
